# Comprehensive Review of Aflatoxin Contamination, Impact on Health and Food Security, and Management Strategies in Pakistan

**DOI:** 10.3390/toxins14120845

**Published:** 2022-12-02

**Authors:** Maryam Ajmal, Wendy Bedale, Abida Akram, Jae-Hyuk Yu

**Affiliations:** 1 Department of Botany, Faculty of Sciences, Pir Mehr Ali Shah Arid Agriculture University, Rawalpindi 46300, Pakistan; 2 Food Research Institute, University of Wisconsin-Madison, Madison, WI 53706, USA; 3 Department of Bacteriology, University of Wisconsin-Madison, Madison, WI 53706, USA; 4 Department of Systems Biotechnology, Konkuk University, Seoul 05029, Republic of Korea

**Keywords:** aflatoxins, agricultural products, Pakistan, food safety and security, control, management

## Abstract

Aflatoxins (AFs) are the most important toxic, mutagenic, and carcinogenic fungal toxins that routinely contaminate food and feed. While more than 20 AFs have been identified to date, aflatoxin B1 (AFB1), B2 (AFB2), G1 (AFG1), G2 (AFG2), and M1 (AFM1) are the most common. Over 25 species of *Aspergillus* have been shown to produce AFs, with *Aspergillus flavus*, *Aspergillus parasiticus*, and *Aspergillus nomius* being the most important and well-known AF-producing fungi. These ubiquitous molds can propagate on agricultural commodities to produce AFs in fields and during harvesting, processing, transportation, and storage. Countries with warmer climates and that produce foods susceptible to AF contamination shoulder a substantial portion of the global AF burden. Pakistan’s warm climate promotes the growth of toxigenic fungi, resulting in frequent AF contamination of human foods and animal feeds. The potential for contamination in Pakistan is exacerbated by improper storage conditions and a lack of regulatory limits and enforcement mechanisms. High levels of AFs in common commodities produced in Pakistan are a major food safety problem, posing serious health risks to the population. Furthermore, aflatoxin contamination contributes to economic losses by limiting exports of these commodities. In this review, recent information regarding the fungal producers of AFs, prevalence of AF contamination of foods and feed, current regulations, and AF prevention and removal strategies are summarized, with a major focus on Pakistan.

## 1. Introduction

Aflatoxins (AFs), first discovered in 1960, are a group of naturally occurring and structurally related toxic, mutagenic, and carcinogenic secondary metabolites produced by certain *Aspergillus* species [1,2]. Numerous (but not all) *Aspergillus* species produce AFs, but *Aspergillus flavus* and *A. parasiticus* are most associated with AF contamination in fields and storage environments [3,4]. Of the more than 20 known AFs, the most common and important are AFB_1_, AFB_2_, AFG_1_, and AFG_2_ [5,6]. AFM_1_ and AFM_2_ are the hydroxylated derivatives of AFB_1_ and AFB_2_, respectively, that are found in milk, milk products, and meat [7,8]. The prevalence of AFB_1_, AFB_2_, AFG_1_, AFG_2_, and AFM_1_ in food makes them more important than the other AFs [9]. Among these, the most frequent and pervasive food and feed contaminant in the world is AFB_1_ [10]. This toxin is of particular concern due to its impact on both human and animal immune systems, as well as its ability to induce cancer [2]. Bioactivated AFB_1_ can bind to DNA, causing G to T transversions. The human tumor suppressor *p53* gene is a primary target of the AFB_1_-DNA adduct [11], which led the International Agency for Research on Cancer to classify AFB_1_ as a Group 1 human carcinogen [12].

Humans may be exposed to AFs through the consumption of AF-tainted foods or the ingestion of foods produced from animals previously exposed to AFs [13]. Chronic dietary exposure to AFs causes substantial health problems in both humans and animals, including slowed development and feeding efficiency, impaired liver and kidney function, weakened immune systems, and other serious disorders [14,15,16]. Chronic exposure to AFs has been estimated to threaten more than half of the world’s population (over 4.5 billion people), including many who live in developing countries [14,17,18]. The numbers of those affected may rise, with increased consumption of AF-contaminated foods expected due to political instability [19], as well as the supply chain challenges and socio-economic hardships caused by the ongoing coronavirus pandemic (COVID-19) [20]. The coronavirus pandemic has also made individuals more susceptible to *Aspergillus* fungi itself, which may cause severe lung infections (pulmonary aspergillosis) in those who are immunocompromised or those with prior lung disease. At least 20 cases of pulmonary aspergillosis linked to coronavirus illnesses have been recorded globally [21]. 

The most important disease related to AF consumption is hepatocellular carcinoma (HCC), also known as liver cancer. HCC is the sixth-most prevalent cancer among men and women of all ages and the fourth leading cause of cancer-related deaths worldwide [22]. According to the data from the Global Cancer Observatory, AFs are thought to be responsible for 4.6–28.2% of all hepatocellular carcinomas worldwide [23]. Developing countries bear most HCC cases (82%) [24], with many new cases occurring in China, West African countries, sub-Saharan Africa, and developing countries in Asia [25]. Between 1970 and 2011, the prevalence of HCC cases in Pakistan increased dramatically, with much of this increase associated with viral hepatitis [26]. Exposure to AFs can act synergistically with viral hepatitis (both hepatitis B and C) in the development of HCC, with the risk of cancer increasing 12-fold when AF exposure occurs in an HBV-infected individual [27]. Carriers of HBV have been shown to have a reduced ability to detoxify AFs, so the relatively high prevalence of HBV carriage (3–5%) in the Pakistani population makes AF exposure an even bigger concern [14]. In Pakistan, liver cancer cases have been associated with AF-contaminated food [28]. A survey conducted in Karachi, Pakistan’s largest city, linked AF contamination in foods to a high prevalence of liver cancer [29]. More recently, a 2021 study found biomarkers of AF exposure in 11% of children in the Multan district of Pakistan, with many of those children also having elevated serum hepatic enzyme levels. The high levels of HBV carriage combined with high rates of AF exposure in Pakistan portend that HCC will continue to be a serious problem [30].

In addition to cancer, AF exposure in humans and animals can lead to aflatoxicosis, which can be an acute or chronic toxicity from consuming (or inhaling) AFs such as those found in contaminated foods. Chronic aflatoxicosis results from lower exposures over a long period and may be manifested by stunted growth, cirrhosis, or hepatocellular carcinoma. Acute aflatoxicosis results from short-term, high-level exposure to AFs, and is characterized by nausea, vomiting, abdominal pain, and other signs of severe liver damage. Acute aflatoxicosis leads to death in approximately 25% of the cases, with intoxicated children more likely to die [14]. The largest recorded aflatoxicosis outbreak occurred in 1974 in western India, resulting in 397 confirmed cases and 106 casualties [31,32]. In Pakistan, Umar et al. [33] reported an outbreak of 45 cases of aflatoxicosis in a bovine herd in Okara, Punjab that had been fed contaminated corn-rich forage. Death occurred in five calves within 3 h of feedstuff ingestion. Laboratory testing of feed samples demonstrated the prevalence of *A. flavus* and *A. parasiticus*, while AFB_1_ levels were as high as 33,500 ppb.

In developing countries, approximately 40% of human productivity is estimated to be lost due to diseases caused or aggravated by AF contamination [14]. Furthermore, AFs are responsible for significant economic losses and have an important impact on international trade. In the United States, annual losses due to mycotoxins (including loss of crops, food animals, and mitigation efforts) are estimated to be as high as USD 1.6 billion, with AFs accounting for a significant proportion of these losses [34]. Although economic loss data for other regions, including Africa and Asia, are less well documented, the losses in these regions are also expected to be high [35]. With many countries having regulations limiting AF contamination levels for imports (discussed later in this review), losses related to agricultural exports are a significant concern for many developing countries such as Pakistan. 

Owing to global climate change, AFs are an emerging threat in regions that were previously free from this menace. Recently, there have been reports of AFs in regions of Europe not previously impacted by AFs [36,37]. Similarly, in the United States, the occurrence of AFs in food is generally uncommon. However, from 2004 to 2013, there were 18 reports of food and feed recalls due to AF contamination in the U.S., with most of these recalls related to dog feed [38]. AF contamination is most common in Asia and Africa, where climatic conditions favor the proliferation of aflatoxigenic strains in fields and during storage [39]. Pakistan has a tropical to subtropical climate, with a mean high temperature of 23.9 °C and annual rainfall of approximately 489 mm. Most (65%) of the annual rainfall is concentrated from June to August during the monsoon season. The hot and humid climate of Pakistan is ideal for mold growth and AF production [40]. The consequences of climate change in countries with hot climates, such as Pakistan, may be serious. However, at high temperatures that inhibit fungal growth, it is possible that mycotoxin levels may be reduced. A prerequisite in determining the effect of climate change is obtaining baseline data of AF concentrations in crops [41]. 

## 2. Fungal Producers of Aflatoxins

*Aspergillus* is a group of widespread and ubiquitous filamentous fungi that exist in a broad variety of environments including air, soil, water, plant debris, manure, animal feed, rotten vegetation, bagasse litter, and indoor air [42,43]. These fungi are commonly found in wheat, rice, corn, peanuts, and oilseed crops, as well as in various agricultural crops before harvesting or during storage [44]. *Aspergillus* species are isolated from various climatic zones, but are more generally found in warm climates between latitudes 16° and 35° and are not widespread above 45° latitude [45]. *Aspergillus* species are commonly found in terrestrial habitats, where they grow as saprophytes on decaying vegetation, in the form of conidia (asexual spores) and sclerotia (winterizing structures) in soil, and in the form of mycelia (vegetative cells) in plant tissue [44]. These molds are very important for the decomposition process, driving the carbon cycle and playing a vital role in the recycling of nutrients [46]. Their conidia can easily be distributed with the help of air movements and by insects [47].

Due to the natural occurrence of *A. flavus* in soil, pre-harvest AF contamination of field crops is prevalent. *A. flavus* also causes post-harvest AF contamination during storage because it spoils the food grains [48]. Crop contamination initiates with the invasion of *Aspergillus* [49]. Fungal contamination and AF production in crops is primarily determined by environmental factors, the ecological make-up of an environment, and the type of crops [50]. AFs are not produced by all *Aspergillus* species and not all species invade all agricultural crops. Therefore, the fungal ecology of the production field determines the levels and severity of AF contamination of agricultural products [51]. Approximately 13 enzymatic reactions involving 30 genes are associated with AF biosynthesis [52]. To date, 28 species of the genus *Aspergillus* have been reported to produce AFs [53,54]. The most important and well-known AF-producing species in foodstuffs are *A. flavus*, *A. parasiticus*, and *A. nomius* [55,56]. While *A. flavus* predominantly produces AFB_1_ and AFB_2_, *A. parasiticus* and *A. nomius* can produce AFB_1_, AFB_2_, AFG_1_, and AFG_2_ [57]. Although it is widely assumed that *A. flavus* is unable to produce type G AFs, some strains have been discovered to be capable of producing both AFG_1_ and AFG_2_ [53]. Recently, Lanier et al. [58] reported that AFM1 is naturally produced by the toxigenic *A. flavus*. Some other *Aspergillus* species, such as *A. pseudotamarii*, *A. togoensis*, *A. agricola*, *A. toxicus*, *A. niger*, *A. ostianus*, *A. ochraceoroseus*, *A. ochraceus*, *A. wentii*, and *A. ruber*, can also produce AFs [59,60]. 

Various physical, nutritional, and biological factors affect the production of AFs by *Aspergillus* fungi [61]. Physical or environmental factors include temperature, pH, relative humidity, light, and levels of atmospheric gases [62,63]. Although *A. flavus* may thrive in a wide range of temperatures ranging 12~48 °C, the optimum temperature for its growth is 28~37 °C [64]. AFs can be produced at a variety of temperatures, although the ideal range for AF production is 25–35 °C [65] but varies between aflatoxins. For example, AFB production is higher than AFG at high temperatures but equal at low temperatures [66]. AF-producing fungi can grow at a wide range of pH (1.7–9.3), but the optimum pH is 3–7 [67]. Fungal growth is decreased at lower pH (3 > pH > 1), while weakly acidic conditions (6 > pH > 3) promote both fungal growth and AF production [68,69]. The ideal relative humidity for the synthesis of AF is greater than 85%, with a relative humidity of 95% or more significantly boosting AF production [70]. The presence of light also has an impact on the proliferation of fungi and the formation of AFs. AF synthesis is inhibited by light, whereas it is increased by darkness [71]. The formation of AFs is also influenced by the availability of O_2_ and CO_2_. A greater CO_2_ concentration and a lower O_2_ concentration hinder the formation of AFs [72].

Numerous nutritional parameters have a significant impact on the production of AFs. A substrate rich in carbohydrates supports a higher level of AF formation because carbohydrates readily offer the carbon that is necessary for effective fungal development and secondary metabolism. Glucose, ribose, sucrose, xylose, and glycerol act as efficient substrates. In contrast, peptone, lactose, and sorbose do not facilitate the production of AFs [73]. *A. flavus* produces more AFs when nitrogen is present in the form of nitrite and nitrate [74]. AF synthesis is further aided by the presence of vitamins, amino acids (glycine, glutamate, and alanine), and various bivalent metals such as zinc and magnesium [75]. Biological factors affect AF production, including conditions that stress crops (weeds, insect injuries) and the presence of fungal species. Weeds compete and cause plant stress, which is linked with elevated AF production. Insect damage causes plants stress and provides sites for aflatoxigenic fungal infection [76]. AF production also depends on the fungal strains present; for example, one study found that only 42 of 55 *A. flavus* strains isolated from oils seeds produced AFs [77]. 

## 3. Regulation of Aflatoxins in Foods and Animal Feeds

**Figure 1:**
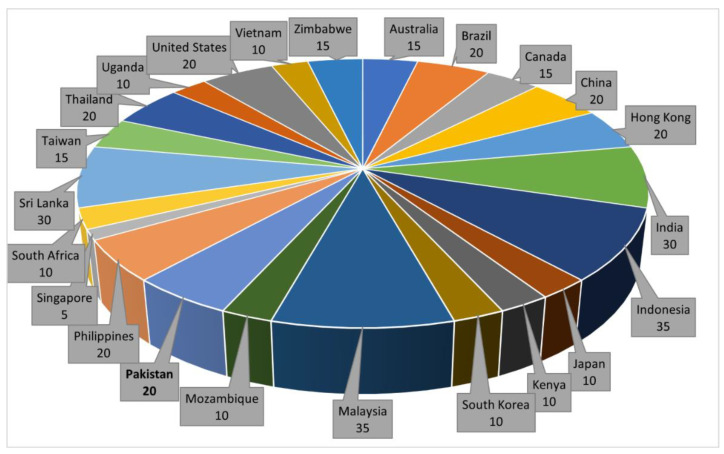
Maximum tolerance limit (MTL) of AFs in food worldwide [82,83,84,85,86,87,88,89,90,91,92,93,94,95,96].

AFs are a significant food safety issue, posing health dangers to humans and animals. Therefore, international agencies, countries, and regions have enacted regulations to minimize the levels of AFs in food and feed [14]. Such levels are typically set by specialist national and multilateral agencies such as the Joint FAO/WHO Expert Committee on Food Additives (JECFA), the European Food Safety Authority (EFSA), the Ministry of Health of the People’s Republic of China, the Food and Drug Administration (FDA) in the United States, and the Pakistan Standards and Quality Control Authority (PSQCA) [78,79]. Globally, approximately 120 countries have laws or legislation specifying allowable limits of AFs in human food and animal feed, but the thresholds permissible differ between countries [45,80,81], as seen in Figure 1. Maximum levels allowed in foods or feeds for different countries also differ depending on the type of product and the import/export systems.

The limit between countries can differ considerably; the maximum/action limit for total AFs has been set at 20 ppb (μgkg^–1^**)** in the United States [97] and 4 ppb in the EU [98]. The PSQCA is responsible for developing and enforcing food standards in Pakistan and has set the maximum acceptable limit for AFs in food and food products at 20 ppb except for milk (whole and skim), which has a maximum limit of 10 ppb. Despite Pakistan’s establishment of maximum tolerated levels of AFs in foods, European and other countries have banned the import of various Pakistani commodities due to poor quality related to AF levels. High levels of AF contamination were found in custard powder and peanut snacks shipped from Pakistan to South Korea and the United Kingdom, respectively. The PSQCA was criticized in both incidents for failing to monitor high AF levels in the food destined for export. For EU member states, the presence of AFs in hot chili powder from Pakistan has also been a cause for concern [99].

The Pakistan Agricultural Research Council (PARC) reported that various countries have rejected considerable quantities of food in recent years due to contamination concerns [99]. AF contamination was the most frequent cause of border rejections by the EU, US, and Australia from 2002 to 2008 (in 35% cases), followed by product composition and prohibited food additives [99]. Since AFs are significant food contaminants harming a nation’s trade, national authorities should pay a special attention to AF control. 

Unfortunately, strict regulations for AF control in food are not always the best option. The fact that high-quality food products are exported and those contaminated with AFs are left to the domestic population may have a negative influence on the health of local customers in Pakistan. AF exposure in low-income populations may increase because contaminated products are sold at reduced prices in local marketplaces. Pakistan, like other less developed nations, has laxer AF enforcement, putting the local population at greater risk of AF exposure from low-quality imported food products. Care must be taken to ensure that strengthening AF standards for exported products does not jeopardize the health of the local population by forcing them to eat contaminated food items that have been rejected.

## 4. Aflatoxin Contamination of Agricultural Products and Foods in Pakistan

In Pakistan, agriculture contributes approximately 21.8% to the GDP (gross domestic product), a substantial portion of the country’s overall economy. More than 65–70% of the population in Pakistan depends on agriculture for its livelihood. The warm, humid environmental conditions of Pakistan are very favorable for the invasion of mycoflora such as *Aspergillus* that can produce AFs as secondary metabolites [100]. High humidity and insufficient ventilation in agricultural commodity storage areas are also problems in Pakistan and are key contributors to *Aspergillus* growth and the production of AFs in foods and feeds [101]. Pakistani crops are, therefore, prone to contamination by AFs, with improper agronomic and storage practices by farmers and processors exacerbating the problem [102,103].

### 4.1. AF Contamination in Cereals

Cereals have been a vital source of human nourishment for thousands of years due to their excellent nutritional qualities and availability [104]. It is the staple food for a large portion of the world’s population [105]. Wheat provides up to 14.1% and 24.3% of the total calorie intake in America and Asia, respectively, while rice alone provides up to 28.5% of total calorie intake in Asia [106]. In the year 2022, estimated cereal production is 2799 million tons worldwide, with a high proportion of coarse grains, wheat, maize, and rice [107]. Wheat (*Triticum aestivum* L.), rice (*Oryza sativa* L.), and maize (*Zea mays* L.) are the major food grain crops of Pakistan. Wheat is grown on 9.2 million hectares (almost 40% of the country’s total cultivated land), with an annual production of 27 million metric tons (MMT) [107]. Rice ranks second among Pakistan’s primary food grain crops, with an annual yield of around 8.2 MMT [107]. During the summer or “Kharif” season, rice is grown on around 10% of Pakistan’s total agricultural land [107]. Pakistan is a major exporter of rice, exporting more than 4 MMT to East Africa, Europe, the Middle East, and China each year [108]. Rice exports are a major source of foreign exchange earnings, accounting for 6.11% of total agricultural value and 1.4% of total GDP [109]. Maize is the third-most important crop after wheat and rice in Pakistan, with an annual production of over 7.8 MMT [107] and accounting for 0.5% of the GDP of Pakistan [110]. It is a multipurpose crop in Pakistan, used as food and animal feed [111]. 

Many studies from Pakistan have reported contamination of AFs in cereal crops, as summarized in Table 1. Cereals are particularly vulnerable to AF contamination. Cereals such as rice and maize are usually grown during hot weather and harvested during the humid summer in Pakistan, conditions generally favorable for AF production. Cereal grains and their flours are hygroscopic and require careful moisture control during storage to prevent AF contamination [112,113]. As shown in Table 1, AF contamination was found in 2~53% of Pakistani wheat samples in five studies, with between 0% and 45.8% of AF contaminated samples having AF levels exceeding EU maximum tolerated levels (MTLs). Even higher percentages of rice were contaminated, with up to 95.4% of samples exhibiting AF contamination. Levels of contamination varied substantially across studies. Previous studies reported that AFB_1_ and AFB_2_ were present at the highest levels in broken rice from Punjab, followed in order by brown rice, white rice, and parboiled rice. Similar results were reported by Lutfullah and Hussain [114]. In a study conducted by Iqbal et al. [115], AF contamination was found in 50% of broken rice samples, a sign that broken rice may be more vulnerable to AF contamination. However, across the studies referenced in Table 1, such trends were not consistently observed. 

Previous studies reported that 27.69–100% maize from various areas of Pakistan were contaminated with AFB_1,_ as shown in Table 1. Overall, across all commodities, 14~100% of samples had AFs levels exceeding the MTLs of the EU.

### 4.2. AF Contamination in Edible Oilseed Crops

Peanut (*Arachis hypogaea* L.), also called ‘The King of Oilseeds’, is one of the most important leguminous oilseed crops grown in Pakistan. Its kernel is rich in oil (43–55% of total content) and protein (25–28%) [140]. Pakistan is the leading peanut producer in the world, with an annual production of 6.10 million tons in 2017, cultivated in an area of approximately 1.33 million hectares (ha). The Pothohar plateau in Punjab province is famous for peanut cultivation, with total annual exports of over 0.1 million tons (USD 15 million) [141]. Peanuts are at high risk for contamination with mycotoxins, particularly AFs, because they are prone to fungal attacks when drying in the field after uprooting [142]. High concentrations of AFs are found in oilseed crops and edible oil products as shown in Table 2 [143]. Mean total AF levels from local peanut oils from two Pakistan markets were 14.52 and 8.59 ppb [144,145], with 35% of samples in the Peshawar market having total AF levels exceeding the EU’s MTL.

Sesame (*Sesamum indicum* L.) is another important oilseed crop of Pakistan, cultivated in an area of approximately 176,000 ha, yielding an annual production of approximately 35,000 metric tons [109]. Sesame seed is also an important source of edible oil that is largely used as a seasoning [103]. In Pakistan, sesame seeds are a common part of cuisine, used regularly in bakeries, confectioneries, and Unani herbal medicines. The sesame crop is vulnerable to a wide range of infectious plant pathogens that damage the plant and facilitate fungal infection (*Fusarium*, *Alternaria*, *Penicillium,* and *Aspergillus)* and mycotoxin production [151]. Only limited studies have reported levels of AF contamination in sesame seeds grown in Pakistan (see Table 2). Ajmal et al. [150] reported high contamination levels for AFB_1_ in sesame seeds from rainfed and irrigated zones of Punjab, Pakistan. In samples from the rainfed zones, 20 ppb were found in 20% of fresh and 100% of stored seeds samples, while in samples from the irrigated zones, 28% of fresh and 60% of stored samples contained AFB_1_ levels more than 20 ppb. Such levels surpass the maximum limits for human consumption assigned by the U.S. FDA and the Food and Agriculture Organization of the United Nations. In another study, Ajmal et al. [152] confirmed that most (72.31%) of 260 isolates of *A. flavus* from sesame seeds grown in Pakistan were aflatoxigenic.

### 4.3. AF Contamination in Nuts and Dried Fruits

Nuts and dried fruits are widely grown and processed in Pakistan. With a total production of 455,990 metric tons (MTs) of dates and 2889.38 MTs of nuts in 2019, Pakistan was placed 6th and 50th in the world, respectively, in their production [153]. Among the dried fruits produced in Pakistan, the government has explicitly prioritized dates as a subsistence crop in vast desert areas [154]. Due to its ideal climatic conditions and very fertile plains, the Gilgit-Baltistan (GB) of the Khyber Pakhtunkhwa (KPK) province is a favorable region to produce dried fruits. In GB, dried fruit is prepared and preserved by removing the original water content by sun drying. The fruits are commonly contaminated by molds during the drying process, which can lead to subsequent AF contamination during storage. However, due to the challenging terrain and remoteness of this region, there is little investment in adequate storage or processing facilities, and 40% of samples of dried fruits from this region showed AFB_1_ levels exceeding the EU’s MTL [155]. Some studies reported high AF contamination levels of dried fruits in the range of 0.05–50.5 ppb, as shown in Table 3.

AFB_1_ occurrence in dried raisins, figs, and dates were reported by Alghalibi and Shater [157]. Asghar et al. [158] evaluated 624 samples of dried fruits for the presence of AFs and reported that 165 (26%) samples were contaminated, with levels ranging from 0.22 to 30.11 ppb and a mean level of 0.85 ppb. They stated that 28 (4%) of the samples were found to exceed the EU limit (4 ppb). Other research revealed that there were significant amounts of total AFs in dried fruit products, with a mean level of total AFs of 2.90 ppb in watermelon seed samples [159]. Masood et al. [160] examined 307 samples of Pakistani edible nuts and dried fruits and found that 132 (43%) of the samples were contaminated with AFB_1_ and total AFs.

### 4.4. AF Contamination in Spices

Roots, bulbs, rhizomes, stems, bark, leaves, and seeds are all utilized to make spices. Spices have substantial economic value and are a common component of many people’s daily diets around the world. Chilies constitute 16% of the global spice trade, ranking the second among spices [161]. Pakistan is the world’s fourth-largest chili producer, after India, China, and Mexico, with an annual production of 141,500 tons cultivated on an area of approximately 157,800 acres. Pakistan ranks 6th worldwide in the export of chilies, exporting 25,000 tons which contribute 1.5% to the country’s total GDP [153]. Chilies, both in fresh and dried form, are considered a basic ingredient of everyday food in Pakistan and are, therefore, used throughout the year. However, they are grown seasonally, harvested in mid-July up to the end of November. 

Due to their plant origin, spices can become contaminated by microbes before, during, and after harvest [162]. In Pakistan, spices are typically harvested, dried, stored, and processed using substandard methods in warm and wet environments that promote the growth of molds. *Aspergillus*, *Penicillium*, and *Rhizopus* are the most common genera of fungi found in spices [163,164,165]. AF contamination of spices is an important problem in Pakistan. Previous studies reported high contamination of AFs in spices from Pakistan, with a range of 2.86–243 ppb as shown in Table 4. Sahar et al. [166] reported that high AF contamination levels were attributed to the drying process rather than pre-harvest conditions. On-farm sun drying is a common practice in Pakistan for various commodities, including chilies, which are typically spread out in open fields for sun drying, then stored under subpar conditions that allow the growth of molds and the production of mycotoxins [167]. Sun drying of the harvested chilies reduced the average moisture content from 69.70% to 9.87%, but also led to a gradual increased level of AFs.

Additionally, the spice markets’ hygienic standards are exceedingly poor in Pakistan, especially in the Karachi district of Sindh. Most of Karachi’s population is from a lower socioeconomic background and is ignorant of AF contamination in spices. When kept in moist, humid conditions for a long time, unpackaged ground spices are a favorable medium for the growth of fungi. Packaging can also influence AF contamination; hot pepper samples in jute bags (common in Pakistan) were reported to be more vulnerable to AF contamination than samples that were packed in polyethylene bags [175]. Loosely packaged compound spices sold in wholesale markets may be easily contaminated by dust, sewerage, and animal or human excrement [176]. Akhund et al. [169] reported the level of AFs in red chilies from the Sindh province, Pakistan. They examined AF levels using TLC and HPLC and demonstrated that 67% of samples were tainted with AFB_1_, with a range of 1.2–600 ppb and a mean level of 131.7 ppb, as shown in Table 4. In another study, spices from different markets of Peshawar were reported to have AF levels ranging from 1.86 to 7.46 ppb. Coriander, omam seed, and turmeric samples contained high levels of AF [102].

### 4.5. AF Contamination in Animal Feeds

Different feed ingredients in Pakistan are susceptible to mold growth due to inadequate harvesting, handling, storage, and processing conditions, so the feedstuff can be contaminated with AFs [177]. As a result of the negative impacts of tainted animal feed, the livestock sectors suffer substantial economic losses. AFB_1_ levels in poultry feed samples from Rawalpindi’s Poultry Research Institute and from West Central Pakistan were reported to be above the safe limit (20 ppb) by Bhatti et al. [178] and Rashid et al. [179], respectively. AFB_2_ was found in poultry diets and feed ingredient samples from Punjab at concentrations ranging from 10.80 to 39.20 ppb [180]. Alam et al. [181] examined 216 samples of chicken feed ingredients (maize, wheat, rice, and cottonseed meal) gathered in the summer, winter, autumn, and spring seasons of 2007/2008 from Swat, Peshawar, and D. I. Khan districts of KPK, Pakistan. Contaminations levels of AFB_1_, AFB_2_, AFG_1_, and AFG_2_ were up to 191.65, 86.85, 167.82, and 89.90 ppb, respectively. The highest concentrations of AFB_1_, AFB_2_, and AFG_2_ were found in the summer samples, while the highest concentration of AFG_1_ was found in the autumn samples. Approximately 61% of samples of poultry feed were tested positive for AFB_1_ by Khan et al. [182], and 47% by Anjum et al. [183]. High levels of AF contamination were found in bakery trash (724.6 ppb) and cottonseed cake (600.8 ppb), as reported in Yunus et al. [184]. A summary of recent studies reporting AF contamination in animal feeds from Pakistan is found in Table 5. Summia et al. [185] reported the highest mean total AF levels in bovine feed collected from Lahore was 229.7 ppb. The mean AFB_1_ level was reported in a range of 3.04–214.9 ppb, as shown in Table 5. 

Fungal infestation is highly affected by the season [137]. In Pakistan, variations in feed AF contamination have been linked to persistent relative humidity and the rainy season, particularly the hot monsoon season, which typically lasts from June to September [179,183,196]. Humidity is strongly linked to the production of AFs in feed during the winter, spring, and summer seasons [191,197,198]. Crops, particularly corn and cotton, are most impacted by AF contamination during the rainy season (June to September) [191]. Corn harvested during the rainy season had a higher level of AFB_1_ (66.4 ppb) than corn harvested during the dry season (37 ppb; Tangendjaja et al. [199]), although these findings are slightly different from those reported by Chauhan et al. [200], which found the highest AFB_1_ levels from June to November and the lowest from December to May. 

Maize and cotton seed cake are important feed ingredients in Pakistan, either directly or as a component of concentrate feed. Rainfall and the accompanying hot and humid conditions during harvest time increases the likelihood of contaminated feed ingredients. Additionally, contamination of these feed materials with AFs can occur when they are kept in storage for subsequent use. Maize crops can be contaminated with a variety of different AFs [201]. Anjum et al. [183] and Bhatti et al. [178] reported the highest contamination of AFs in corn. These results are consistent with those of Kamkar et al. [202], who found that increased moisture content and delayed storage caused increased AFB_1_ contamination in animal feed. The direct link between AFB_1_ contamination and storage period was reported in numerous previous research [203,204,205]. Reddy and Salleh [206] reported that 23% of animal feed samples were contaminated with AFB_1_ at levels ranging from 21 to 135 ppb. Similarly, Anjum et al. [183] reported that 61% of maize AFB_1_ contaminated samples exceeded the permissible limits. Additionally, farmers feed scraps of bread, a significant source of AF contamination, to animals. According to Asi et al. [207], animals fed on bread pieces and concentrates produced more AF in their milk. 

Due to the shortage of available feed during the winter, farmers supplement animal feed with compound feed. Compound feed is typically made from leftover grains, making it especially vulnerable to rises in AF levels during storage. According to Asi et al. [207], animals in Pakistan that were typically fed with compound feed showed higher concentrations of AFM_1_ in their milk than animals that grazed or were fed fresh green feed. To ensure the highest milk production during the winter, when fresh pasture or fresh feed is not available, farmers feed animals the highest possible amounts of corn, cotton seeds or cotton seed cake, raw rice bran/rice polish, wheat bran, and gluten. These components support a high level of milk production but are most susceptible to fungal infestation and AF production [208,209].

Crop harvesting time is another factor that has been connected to increased levels of AF contamination in winter. In Punjab, Pakistan, the corn-harvesting time is October, while the cotton-harvesting time is August to September, so corn and cotton seed become a primary, economically efficient source of feed in these months. Dairy farmers grow these crops and feed animals without knowing whether the feeds are contaminated with AFs [207,210,211,212].

### 4.6. AF Contamination of Milk 

The livestock sector is important for the economic development of all countries. It plays a crucial role in reducing the poverty of rural areas by providing food and income [213]. Milk is a rich source of nutrition for all age groups that contributes to the optimal growth of newborns and children. Pakistan produces over 60 billion liters of milk annually, making it the world’s fourth-largest milk producer [214]. It contributes 46.8% of agriculture revenue, where 10–25% of the income is generated by rural people [215]. As the demand for milk rises, it becomes more difficult for the dairy industry in developing nations to maintain a uniform and standardized quality. In Pakistan, milk demand is fulfilled primarily (94%) by informal, nonindustrial supplies, whereas the packed milk sector bridges only 6% of the gap. Milk in this informal supply chain is expected to have high levels of AFM_1_ contamination [216].

AFs such as AFB_1_ are bio-transformed into AFM_1_ in dairy animals’ livers and subsequently excreted into milk, eventually reaching the humans who consume the milk [217,218]. Animals fed on AF-contaminated feed exhibit decreased growth rate, decreased milk production, and lowered milk quality, in addition to compromised immunity against infections [219]. 

AFM_1_ in raw milk cannot be destroyed by pasteurization, heat processing, or other simple methods [220]. Recent research has emphasized significant human health risks connected to the consumption of milk tainted with AFs [221,222]. AFM_1_ contamination of milk is a global issue, especially in developing countries. The Punjab province of Pakistan is the major cash-crop-producing and livestock-keeping area. AFM_1_ contamination screening of the milk from areas within Punjab found that the average AFM_1_ contamination levels were above US and EU regulatory limits [223] Table 6. Similarly, 99% of fresh milk samples in India (which neighbors Pakistan) exceeded the Codex limits [205]. 

In Pakistan, particularly in Punjab, the months of December to March are associated with increased rates of milk contamination [191]. In the winter, when green fodder is scarce, farmers are forced to use stockpiled feed sources [191]. As a result, milk from cows that consume stored feed is positively associated with AFM_1_ [206,207], and various studies have reported significantly higher AFM_1_ contamination levels in milk during the autumn or winter season in Pakistan [134,186,224,225,226]. High AFM_1_ contamination levels in raw milk samples were reported during the winter and autumn seasons, with average values of 54.24 and 34.92 ppt (parts-per trillion: ng/L), respectively, according to studies by Shokri and Torabi [212]. Akbar et al. [216] reported that milk samples had the highest range of AFM_1_ contamination in November and the lowest in May. From March to August, the AFM_1_ contamination levels of the milk samples rapidly declined in all locations. AFM_1_ trends were nearly identical across all locations in Pakistan, i.e., AFM1 levels in milk produced from mid-April to August typically meet the US MRL. Following August, a marked rise in the concentration of AFM_1_ is found in all local milk samples. The most and least contaminated samples were found in the months of February and July, respectively [216]. 

**Table 6:** Occurrence of AFs in milk from 2008 to 2022.

Commodity	Area of Collection (Year)	AFs	Analytical Method	Contaminated Samples/Total Samples (Incidence %)	Mean	Range	Over EU MTL (%) *	References
Raw milk	Punjab, Pakistan (2017–2018)	AFM_1_	LC	134/278 (48.2%)	70.5 ng/L	–	17.3	Waqas et al. [189]
Branded milk Non-branded	Lahore, Punjab (2016–2017)	AFM_1_ AFM_1_	ELISA ELISA	23/40 (58) 40/40 (100)	225.2 ppt 828.4 ppt	54.30–577.9 ppt 17.34–2735 ppt	58 95	Zahra et al. [227]
Raw milk	Dairy farms, Punjab (2015)	AFM_1_	ELISA	200/240 (83.33)	0.59 µg/L	–	53*	Akbar et al. [191]
Raw milk Processed milk	Peri-urban dairy farms (2016)	AFM_1_ AFM_1_	ELISA ELISA	372/372 (100) 45/45 (100)	3164 ng/L 558.1 ng/L	LOD–15994 ng/L 7.3–3935.5 ng/L	93.3 66.7	Yunus et al. [184]
Raw milk	Quetta	AFM_1_	ELISA	88/100 (88)	257 ng/L	236.6–292.9 ng/L	100	Fahmid et al. [228]
Raw milk Processed milk	Punjab (2013–2015)	AFM_1_ AFM_1_	ELISA ELISA	294/340 (86.6) 152/230 (66.7)	0.52 ng/mL 0.13 ng/ml	0.17–1.63 ng/mL 0.01–0.95 ng/mL	34.45 * 16.66 *	Tahira et al. [229]
Raw milk	Punjab, Pakistan (2015)	AFM_1_	ELISA	844/960 (87.9)	0.642 µg/L	*–*	70*	Akbar et al. [216]
Raw milk Raw milk TW (Processed) UHT (Processed)	Lahore, Multan	AFM_1_ AFM_1_ AFM_1_ AFM_1_	ELISA ELISA ELISA ELISA	85/94 (90) 52/56 (92) 30/30 (100) 30/30 (100)	0.232 µg/L 0.139 µg/L 0.113 µg/L 0.164 µg/L	0.006–0.521 µg/L 0.015–0.554 µg/L 0.013–0.257 µg/L 0.010–0.345 µg/L	71 73 56 66	Ahmad et al. [230]
Raw milk Pasteurized milk UHT milk	Islamabad (2016)	AFM_1_ AFM_1_ AFM_1_	ELISA ELISA ELISA	– – –	1535 ng/L 939.5 ng/L 254.9 ng/L	1912–7460 ng/L 32.8–4808 ng/L LOD–1536 ng/L	90 * 55 * 12.9 *	Yunus et al. [224]
Milk product (mithai)	Lahore (2017)	AFM_1_	HPLC	152/200 (76)	–	0.004–1.49 µg/kg	80	Naz et al. [231]
Raw milk	Local market, Karachi (2016–2017)	AFM_1_	ELISA	143/156 (91.7)	346.2 ng/L	20–3090 ng/L	80.1	Asghar et al. [232]
Raw milk UHT milk Powdered milk Flavored milk Yogurt Flavored yogurt	Summer, Punjab (2014–2015)	AFM_1_ AFM_1_ AFM_1_ AFM_1_ AFM_1_ AFM_1_	HPLC HPLC HPLC HPLC HPLC HPLC	19/32 (59.4) 16/25 (64) 9/32 (28.1) 12/25 (48.0) 11/30 (36.6) 10/25 (40)	94.9 ng/L 75.2 ng/L 65.1 ng/L 45.3 ng/L 59.6 ng/kg 45.3 ng/kg	LOD–229.6 ng/L LOD–190.8 ng/L LOD–178.5 ng/L LOD–110.1 ng/L LOD–158.2 ng/kg LOD–102.5 ng/kg	37.5 32.0 12.5 20 20 16	Iqbal et al. [233]
Raw milk UHT milk Powdered milk Flavored milk Yogurt Flavored yogurt	Winter, Punjab (2014–2015)	AFM_1_ AFM_1_ AFM_1_ AFM_1_ AFM_1_ AFM_1_	HPLC HPLC HPLC HPLC HPLC HPLC	29/42 (69) 26/35 (74.2) 12/32 (37.5) 15/28 (53.3) 15/36 (41.6) 17/30 (56.6)	129.6 ng/L 98.5 ng/L 89.7 ng/L 46.4 ng/L 63.6 ng/kg 50.5 ng/kg	LOD–345.8 ng/L LOD–302.9 ng/L LOD–278.4 ng/L LOD–198.3 ng/L LOD–196.3 ng/kg LOD–220.5 ng/kg	38.1 37.1 15.6 21.4 27.7 40	Iqbal et al. [233]
Raw milk	Punjab (2012–2013)	AFM_1_	ELISA	468/485 (96.5)	–	–	87.22 *	Aslam et al. [225]
Raw milk	Punjab (2013–2014)	AFM_1_	ELISA	483/520 (92.8)	0.06 µg/L	0.00–0.26 µg/L	53	Ismail et al. [234]
Raw milk	Sindh	AFM_1_	ELISA	81/84 (96.43)	0.38 µg/L	0.01–0.76 µg/L	70 *	Jawaid et al. [226]
Buffalo milk Cow milk	Faisalabad (2013)	AFM_1_ AFM_1_	HPLC HPLC	– –	0.081 µg/L 0.0655 µg/L	0.037–0.114 µg/L 0.0243–0.1 µg/L	84 72	Sajid et al. [235]
Raw milk	Urban farmhouse, Rural farmhouse, Punjab (2011)	AFM_1_ AFM_1_	HPLC HPLC	38/59 (64) 25/48 (52)	0.064 µg/L 0.04 µg/L	LOD–0.98 µg/L LOD–0.71 µg/L	42 27	Iqbal et al. [236]
Milk Yogurt White cheese Cheese cream Butter	Punjab (2010–2011)	AFM_1_ AFM_1_ AFM_1_ AFM_1_ AFM_1_	HPLC HPLC HPLC HPLC HPLC	76/107 (71) 59/96 (61) 93/119 (78) 89/150 (59) 33/74 (45)	150.7 ng/L 90.4 ng/kg 147.8 ng/kg 102.6 ng/kg 69.7 ng/kg	4–845.4 ng/L 4–615.8 ng/kg 4–595.4 ng/kg 4–456.3 ng/kg 4–413.4 ng/kg	58 47 15 11 52	Iqbal et al. [237]
Raw milk UHT milk Yogurt Butter Ice cream	Central Punjab during summer (2012)	AFM_1_ AFM_1_ AFM_1_ AFM_1_ AFM_1_	HPLC HPLC HPLC HPLC HPLC	20/56 (36) 12/39 (31) 13/45 (29) 14/35 (40) 9/37 (24)	0.028 µg/L 0.021 µg/L 0.019µg/kg 0.015µg/kg 0.012µg/kg	LOD–0.89 µg/L LOD–0.51 µg/L LOD–0.88 µg/kg LOD–0.78 µg/kg LOD–0.34 µg/kg	13 9 8 7 2	Iqbal et al. [186]
Raw milk UHT milk Yogurt Butter Ice cream	Central Punjab during winter (2012)	AFM_1_ AFM_1_ AFM_1_ AFM_1_ AFM_1_	HPLC HPLC HPLC HPLC HPLC	19/48 (40) 23/45 (51) 19/51 (37) 21/35 (60) 18/42 (43)	0.073 µg/L 0.060 µg/L 0.053µg/kg 0.036µg/kg 0.021µg/kg	LOD–0.45 µg/L LOD–0.51 µg/L LOD–0.44 µg/kg LOD–0.57 µg/kg LOD–0.67 µg/kg	27 24 25 34 17	Iqbal et al. [186]
Buffalo milk Cow milk Goat milk Sheep milk Camel milk	Punjab during summer (2009–2010)	AFM_1_ AFM_1_ AFM_1_ AFM_1_ AFM_1_	HPLC HPLC HPLC HPLC HPLC	– – – – –	0.042 µg/L 0.022 µg/L 0.018 µg/L 0.024 µg/L 0.010 µg/L	0.025–0.105 µg/L 0.014–0.095 µg/L 0.009–0.088 µg/L 0.012–0.069 µg/L 0.005–0.081 µg/L	38 33 36 21 14	Asi et al. [207]
Buffalo milk Cow milk Goat milk Sheep milk Camel milk	Punjab during winter (2009–2010)	AFM_1_ AFM_1_ AFM_1_ AFM_1_ AFM_1_	HPLC HPLC HPLC HPLC HPLC	– – – – –	0.091 µg/L 0.089 µg/L 0.069 µg/L 0.079 µg/L 0.058 µg/L	0.050–0.200 µg/L 0.065–0.150 µg/L 0.008–0.090 µg/L 0.010–0.098 µg/L 0.012–0.064 µg/L	55 56 58 32 27	Asi et al. [207]
Raw milk Raw milk Raw milk Sweets	Local shop milk Household milk Dairy farm milk	AFM_1_ AFM_1_ AFM_1_ AFM_1_	ELISA ELISA ELISA ELISA	137/175 (78) 25/40 (62) 15/17 (88) 134/138 (97)	0.176 µg/L 0.47 µg/L 0.11 µg/L 0.48 µg/kg	0.002–1.6 µg/L 0.003–1.9 µg/L 0.002–0.794 µg/L 0.01–1.5 µg/kg	28.6 45 41 78	Sadia et al. [238]
Buffalo milk Buffalo milk Cow milk Cow milk	Punjab KPK Punjab KPK (2009–2010)	AFM_1_ AFM_1_ AFM_1_ AFM_1_	HPLC HPLC HPLC HPLC	22/48 (45.83) 24/46 (52.17) 20/41 (48.78 22/43 (51.16)	0.040µg/kg 0.066µg/kg 0.030µg/kg 0.045µg/kg	LOD–0.137 µg/kg LOD–0.350 µg/kg LOD–0.062 µg/kg LOD–0.084 µg/kg	27 42 30 32	Iqbal et al. [175]
Raw milk	Lahore (2007)	AFM_1_	HPLC	68/84 (81)	17.38 µg/L	0.69–100 µg/L	81 *	Khushi et al. [239]
Buffalo milk Cow milk Goat milk Sheep milk Camel milk	Faisalabad (2005)	AFM_1_ AFM_1_ AFM_1_ AFM_1_ AFM_1_	HPLC HPLC HPLC HPLC HPLC	19/55 (34.5) 15/40 (37.5) 6/30 (20) 4/24 (16.7) 0/20 (0)	0.013 µg/L 0.014 µg/L 0.002 µg/L 0.002 µg/L 0	– – – – –	15.8 20 0 0 0	Hussain et al. [240]
Raw milk	Lahore (2007)	AFM_1_	HPLC	68/84 (81)	17.38 µg/L	0.69–100 µg/L	100	Khushi et al. [239]
Buffalo milk Cow milk	Central areas of Punjab (2007)	AFM_1_	HPLC	153/360 (42) 63/120 (52.5)	0.027 µg/L 0.044 µg/L	– –	0.6 *	Hussain et al. [209]
Raw milk (Dairy animals)	Punjab (2005)	AFM_1_	Immunoaffinity columns and Fluorometer	168/168 (100)	0.371µg/L	0.01–0.70 µg/L	99.4 3 *	Hussain et al. [10]

Abbreviations (AFM_1_ = Aflatoxin M_1_, LC = liquid chromatography, HPLC = high-performance liquid chromatography, KPK = Khyber Pakhtunkhwa, LOD = limit of detection (i.e., 0.004 µg/kg), ppt = parts per trillion, µg/L = microgram per liter, ng/L = nanogram per liter, µg/kg = microgram per kilogram, ng/kg= nanogram per kilogram, ng/mL = nanogram per milli liter, EU = European Union, MTL = maximum tolerance limit). (%) * = Percentage of the samples out of contaminated samples exceeding the maximum tolerance limits (MTL) recommended by European Union [98]. Value * exceeding the exceeding the MTL recommended by FAO and FDA [97].

## 5. Dietary Exposure to Aflatoxins

Two methods are commonly used to determine the human exposure to AFs. The first and most popular approach combines data on food consumption with AF levels found in food samples [241]. By dividing the total AFs consumed by the average human body weight, the exposure is further standardized and represented as nanograms per kilogram of body weight per day (ngkg^–1^ b.w./day) [242]. The European Food Safety Authority (EFSA) has developed detailed guidelines for dietary data collection and processing for risk and exposure assessment purposes [243]. Measuring AF biomarkers in human bodily fluids is an alternate approach that is thought to be a direct and more precise method of determining human AF exposure [244]. Biomarkers of AFs such as the AF-N7-guanine adduct excreted in urine and AFM_1_ present in breast milk are used to determine short-term exposure to AFB_1_, while AF-albumin adduct levels in plasma or serum are used for chronic exposure assessment [245].

Although AFs are genotoxic carcinogens, and regulations regarding their presence in food have mostly focused on its carcinogenic consequences, international risk assessment agencies such as the Joint Expert Committee on Food Additives (JECFA) have never defined a TDI for AF [246]. However, daily exposure to levels as low as 1 ngkg^–1^ b.w./day is considered dangerous to human health [247]. Turna and Wu [246] estimated a range of TDIs for AF-related immune impairment is 0.017–0.082 μg/kg b.w./day.

In Pakistan, average adults consume approximately 171–239 g of rice daily. Abdullah et al. [248] reported the estimated AF intake for typical rice consumers ranged between 19.1 and 26.6 ngkg^–1^ b.w./day. Iqbal et al. [127] reported the average estimated dietary intake (EDI) of AFs for adults in Pakistan was 22.2–ngkg^−1^ b.w./day. These intakes are much higher than 1 ngkg^–1^ b.w./day that is considered unsafe and pose considerable health risks to people in Pakistan [249]. Majeed et al. [123] also reported that the estimated AFB_1_ and AFB_2_ intake in Pakistani adults and children through rice consumption was above the recommended limits. More recently, Xia et al. [116] estimated the average probable daily intake (PDI) and EDI for AFB_1_ to be 30.3 ngkg^–1^ b.w./day and 3.5 ngkg^–1^ b.w./day, respectively, in the rural population of Pakistan. In contrast, Raad et al. [250] reported a lower level of dietary exposure to AFB_1_, i.e., 0.63–0.66 ngkg^–1^ b.w./day for Pakistani adults. 

According to Ismail et al. [234], the people of Pakistan are also at high risk of health issues related to AFM_1_ in milk. The estimated daily intake (EDI) of AFM_1_ during various seasons of the year for various age groups was found in the range of 0.22–5.45 ngkg^–1^ b.w./day, with infants in the highest risk group. Male babies were found to have the highest EDI values for AFM_1_ during the winter (5.45 ngkg^–1^ b.w./day), whereas adult females had the lowest values during the summer (0.22 ngkg^–1^ b.w./day). Children were reported to be more susceptible to AFM_1_ because of higher milk intake levels and lower body weight. EDI values for AFM_1_ decreased with age, with adults having the lowest levels [234]. According to Xia et al. [251], the rural Pakistani population had a high prevalence of AFM_1_ in urine. 

AFM_1_ prevalence in human breast milk samples is a sign that mothers’ diets are contaminated with AFs. AFM_1_ contamination in breast milk samples is ultimately a reflection of AF contamination of Pakistani food products. The range of AFM_1_ in milk samples from women in Southern Punjab was reported to be between 0.001 and 0.044 µg/L by Khan et al. [252], with 6.4% of samples exceeding the EU’s maximum limit (0.025 µg/L).

A recent study in a rural Pakistan population used urinary biomarkers to estimate AFB_1_ exposure and calculate its contribution to liver cancers. Urinary AFM_1_ (a marker of AFB_1_ exposure) was found in 69% of urine samples (mean levels of 0.023 ng/mL, with a maximum level of 0.393 ng/mL) [116]. Compared to the average rates of liver cancer in Pakistan (7.6 and 2.8 cases per 10^5^ individuals each year for males and females, respectively), AFB_1_ exposure calculated via urinary marker data contributes significantly to the total risk (0.514 cases per 10^5^ individuals annually) [253]. This risk was significantly higher than the risk attributed to AFB_1_ exposure from rice alone (estimated by food frequency questionnaires) in Pakistan; 0.07 to 0.122 cancer cases per 10^5^ individuals per year [123]. 

## 6. Control Strategies for Aflatoxins Contamination

AF contamination in the field is difficult to control due to several factors, including temperature, insect infestation, humidity, soil moisture, and mineral deficiencies [254]. Various strategies can be used before or after harvest or during storage to prevent their contamination with AFs [255]. At the pre-harvest stage, AF contamination can be reduced by proper agronomic techniques and using resistant cultivars. In addition, insect and mechanical damages to plants should be minimized during the preharvest stages [256].

**Figure 2:**
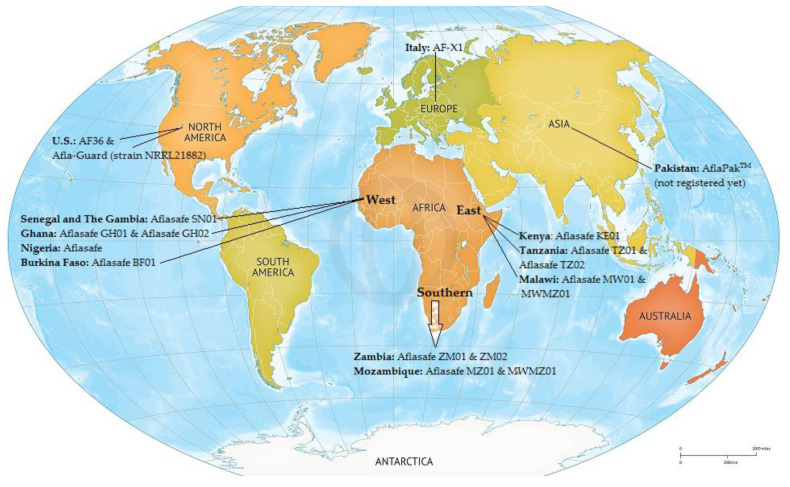
Map of the world showing registered AF biopesticides and utilizing countries [259,260,261,262,263,264,265,266].

An effective biological method to prevent AF contamination in the field and during storage is the introduction of naturally occurring non-aflatoxigenic strains of *A. flavus* and *A. parasiticus* to competitively exclude aflatoxigenic strains. A conidial suspension of a non-aflatoxigenic strains is administered to the soil before planting or directly to the seedlings [257]. Successful management of AF production in the field by using non-aflatoxigenic *Aspergillus* also helps prevent AF contamination during later storage. This strategy was first used by Cotty and Bayman [258] and it has been subsequently employed for the control of AFs around the globe (Figure 2). While biopesticides are not yet used in Pakistan, AflaPak^TM^, a regionally named native biocontrol product, is in development as a public-partnership between the USDA, the Centre for Agriculture and Biosciences International (CABI), Rafhan Maize, and the National Agricultural Research Council (NARC) (https://www.cabi.org/projects/ AF -control-in-pakistan/ accessed on 15 August 2022). AflaPak^TM^ is being developed for the control of AF in maize. CABI is currently evaluating the efficacy of AflaPak^TM^ in seven maize growing districts of Punjab province in Pakistan, hopefully leading to the commercialization and adoption of this biocontrol product in Pakistan. Once registered, AflaPak^TM^ will be the first registered native biocontrol product of a fungal nature in Pakistan, opening opportunities for more green technologies to be adopted in Pakistan.

**Figure 3:**
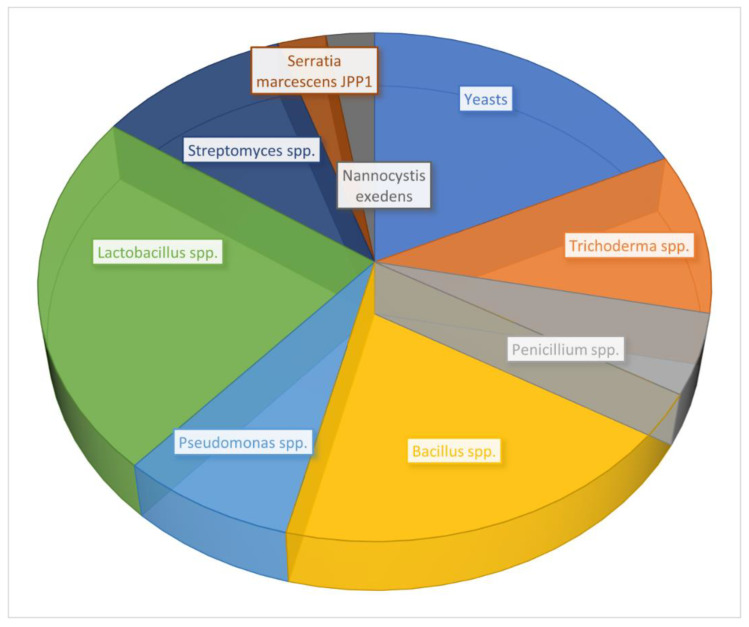
Biological control of AF contamination with microorganisms currently under development [270,271,272,273,274,275].

Some biopesticide (non-aflatoxigenic strains) fungal strains do not produce AFs because of a deletion in the AF biosynthetic gene cluster. The AF36 strain, on the other hand, does not produce AFs due to a SNP (single nucleotide polymorphism) that triggers a stop codon in a key gene involved in the polyketide biosynthesis necessary for AF production [267]. In addition to *Aspergillus*, other fungal species such as yeast, *Trichoderma,* and *Penicillium* have been demonstrated to reduce AFs in the field [268,269]. Globally, some of the microorganisms are currently at the experimental stage against fungi that produce AFs (Figure 3). 

At the post-harvest stage, the dry chain technique has been used to minimize AF contamination of crops in Pakistan. The basic principle of the dry chain is to dry grains to reduce moisture content and to maintain this dryness throughout the supply chain [275]. A dry chain is an economically viable and environmentally friendly method to reduce the chances of AF contamination. Bakhtavar et al. [276] reported that insect populations, associated storage losses, and deterioration of maize seed quality with respect to loss of germination, food reserves, and increased AF contamination can be controlled by maintaining the dry chain through hermetic storage of maize grain at 8% and 10% seed moisture content.

## 7. Detoxification of Aflatoxins

Once a food or food product has been contaminated, AF detoxification is needed. Detoxification involves removing or minimizing the poisonous effects of AF, and can involve physical, chemical, or biological methods. Any detoxification procedure used on human food, according to the FAO, must inactivate, destroy, or remove AFs; not create or leave toxic, carcinogenic, or mutagenic residues on the treated substrates; maintain the nutritional, sensory, or other quality attributes of the food product; and be able to get rid of fungal spores or mycelium that could multiply and produce mycotoxins [277].

### 7.1. Physical Detoxification

Thermal and ultrasonic treatments, solvent extraction, mechanical sorting, adsorption, UV and solar irradiation, density gradient, ozone gas, flotation, gamma rays, electronic eye sorting, roasting, and microwave heating are some of the physical techniques used to inactivate or reduce AF contamination [254]. Damaged and fragmented grains contain higher concentrations of mycotoxins; therefore, removing them lowers the overall contamination [278]. Hand sorting and segregation of grains based on physical characteristics can be beneficial in lowering AF levels in agricultural products, but not on a large scale. Treatment with UV light and ionization can prevent AF contamination in food and extend food shelf life because such treatments degrade the fungal cells [279]. Thermal treatments can also be very effective. Depending on the thermal treatment used and the commodity being treated, amounts of AFs can be reduced by 9% to 100%. For example, when fruits and other species are autoclaved at 120 °C for 30 min, amounts of AFs can be reduced by 9~39%; however, when peanuts are autoclaved at 1.5 atm for 90 min, the amount of AF can be reduced by up to 100% [280]. However, because AFs are heat resistant and not entirely degraded at temperatures commonly used in food processing (80–121 °C), typical cooking procedures such as frying, boiling, or pasteurization are unlikely to result in a significant reduction in AF levels [280]. In addition to thermal processing, non-thermal processes such as cold plasma can be utilized to reduce the AF levels in certain grains and nuts by up to 95% [281]. 

One of the most effective methods to reduce exposure to AFs such as AFB_1_ is the addition of non-nutritive adsorbent in contaminated animal feed to decrease the bioavailability of AFs in the gastrointestinal tract. However, the non-specificity of adsorbents and their high costs are major drawbacks for such a method. In addition, due to their non-degradable nature, which is necessary to survive the gastrointestinal tract, deposition of absorbents in the environment when excreted from animals also limits their use [282].

In Pakistan, some studies have reported detoxification of AFs by using physical methods. A study by Khan et al. [283] reported a 78% reduction in AFs in red chilies by removing midget/dwarfed, damaged, broken, dusty, and dirty chilies. Khan and Zahoor [284] effectively detoxified AFB_1_ in poultry feed by using a novel adsorbent that was prepared from bagasse. Saleemi et al. [285] investigated the effect of a locally produced mycotoxin binder in commercial broiler chicks. This study showed that local toxin binder (bentonite clay 58%, baker yeast 40%, and silymarin 2%) was a more cost-effective way to mitigate AFB_1_ in poultry feed than commercial toxin binders. Summia et al. [286] reported AF reduction of up to 58% by heating poultry feed samples at 250 °C for 10 min. Awan et al. [287] detoxified AFB_1_ in pine nuts by using UV irradiation and reported 50%, 70%, and 90% detoxification after exposure to UV for 20, 40, and 60 min, respectively. Some of these physical procedures, while effective, are expensive and may have an impact on key nutrients or sensory characteristics in the commodities. Gillani et al. [288] reported the use of 15 kGy gamma irradiation for 24 h, sunlight-drying for 20 h, and UV irradiation for 12 h almost completely degraded AFs in maize. Microwave heating for 120 s resulted in 9–33% degradation of AFs in maize. Moreover, the treatment of maize grain extracts with activated charcoal (5% *w*/*w*) removed 96% of total AFs and AFB_1_. The use of bentonite at the same rate removed total AFs and AFB_1_ by 73% and 92%, respectively [288].

### 7.2. Chemical Detoxification

A variety of chemicals have been used to detoxify AFs, including oxidizing and reducing reagents, acids, bases, and chlorinating agents. The efficacy of AF detoxification is increased when chemical agents are utilized in conjunction with physical approaches. More than 100 chemical compounds have been found to inhibit or reduce mold development, resulting in a reduction in AF levels [289]. In Pakistan, Iqbal et al. [141] reported that AFs were reduced by 44% in samples of corn with the addition of a single step of rinsing the corn with water. Nazir et al. [122] reported 80.02% degradation of AFB_1_ in rice and cattle feed by treating the contaminated samples with NaHCO_3_ and washing them with distilled water three times. According to Summia et al. [286], 0.5% hydrochloric acid treatment of feed samples lowered AF levels by up to 58.4%.

Chemical treatments to detoxify AF-contaminated agricultural goods have been effectively used in a variety of contexts, process parameters, and food products, but they may leave some toxic residues in the treated substrate. Detoxification of AFs by using natural substances may be more acceptable to consumers than using synthetic chemicals. In Pakistan, several studies reported detoxification of AFs by using natural substances. Nazir et al. [122] reported that black seed oil reduced AF contamination up to 100% in rice, maize, and corn. Anjum et al. [289] used a water-based extract of *Acacia nilotica* to achieve 86–90% detoxification of both AFB_1_ and AFB_2_ in maize when incubated for 72 h at 60 °C and pH 10, and 82–83% detoxification at 30 °C and pH 8. Nazir et al. [122] detoxified AFs in food by using natural compounds such as sodium bicarbonate, citric acid, extract of *Allium sativum*, and black seed oil. Reduction in AFs was reported to be 63~100%. The same study also reported the 63.59% to 90% reduction in AFB_1_ levels by treating contaminated samples with lemon juice. Awan et al. [287] reported that *Zingiber officinale* powder detoxified 90% of AFs in dry fruits.

While detoxification of AFs has been studied extensively, little is known about the potential of food processing to increase or hide AF exposure. Chemical and physical treatments applied to food may release AFs from masked forms and make them bioavailable or convert into forms not detectable by conventional analytical methods [290], while retaining their toxic potential [291] or stimulating fungi to produce AFs, e.g., during steeping of barley. Analytical tools for mycotoxins transformed by processing by structural modification or binding to the food matrix need to be developed.

### 7.3. Biological Detoxification

Biological methods, in comparison to other methods of AF detoxification, are thought to be less aggressive, environmentally safe, and cost-effective. Such methods involve using microbes and their products to eliminate AFs from food or feed by surface adsorption, degradation into nontoxic chemicals, or binding to inhibit bioavailability [292]. For example, *Flavobacterium aurantiacum* effectively eradicates AFB_1_ from a variety of foods, including oil, milk, peanut butter, maize, and peanuts, without producing any harmful byproducts [293]. Biodegradation technology has provided an appealing alternative for controlling or eliminating AFs while maintaining food and feed quality and safety. Customers are becoming increasingly opposed to the use of chemical and synthetic compounds in their foods; therefore, the employment of biological agents provides a more “natural” appeal [294]. Azeem et al. [295] assessed the in vitro binding of AFB_1_ by probiotic *Lactobacilli* by incubating with a standard amount of AFB_1_ in phosphate-buffered saline at 37 °C for 2 h. The isolates’ levels of AFB_1_ binding ranged from 28 to 65%. Four isolates (PDP 10, PDP 24, PL 120, and PL 149) resulted in complete binding of AFs. 

The use of plant-derived nanoparticles (NPs) is an emerging technology with a potential to reduce AF contamination levels. Various plants leaf extracts such as *Gongronema latifolium, Moringa oleifera,* bitter leaf (*Veronica amygdalina*), and *Psidium guajava* have been utilized for the biosynthesis of metal NPs [296,297]. In Pakistan, some studies have reported antifungal and AF inhibitory activities of green synthesized nanoparticles. Asghar et al. [298] prepared iron (Fe), copper (Cu), and silver (Ag) nanoparticles (NPs) from a *Syzygium cumini* leaf extract. The obtained metal NPs showed excellent antimicrobial activities against *A. flavus* and *A. parasiticus.* Furthermore, production of AFs was also significantly inhibited; no AFs (100% inhibition) were produced when treated with 100 μgmL^−1^ of Ag-NPs in both strains in vitro, while 43−49% and 76−80% reductions were achieved using Fe- and Cu-NPs, respectively.

## 8. Conclusions

This review summarizes AF-producing fungi, agricultural and food products prone to contamination with AFs, exposure and risk assessment of AFs, related legislations, and current AF prevention and mitigation options, with a focus on AF problems encountered in Pakistan. AFs are a significant problem in Pakistan due to the nation’s hot and humid climate and the substantial role of agriculture in its economy. In addition, the high rates of hepatitis viral infections in Pakistani people means that the population is more vulnerable to AF-related cancers. Numerous studies have found agriculture crops and food products produced in Pakistan were contaminated with AFs at levels above the US and EU permissible limits. Exporting food products with high levels of AFs results in trade restrictions. If food goods planned for export have been found to contain high levels of AFs, it can be safely assumed that local consumers are at danger because of the availability of highly contaminated products in local markets that have not been tested for AFs. There is a critical need to raise awareness among the population regarding the occurrence of these carcinogenic toxins in food commodities and their potential risks to humans and animals. Consumption of AF-contaminated feed has a negative impact on livestock performance, including decreased productivity and weight gain. As a result, the livestock industries in Pakistan experience substantial economic losses. Most of the AF-related studies have been conducted in the Punjab province of Pakistan. More comprehensive nationwide investigations are required to properly assess the AF contamination issues more broadly. To compete on the international market, Pakistan must improve compliance to its standards in food quality control related to AFs. The regulatory authorities should take this issue of AF contamination into account for consumer safety and the nation’s economy, and proper control measures should be implemented and the food quality control system upgraded.

## Figures and Tables

**Table 1:** Occurrence of AFs in cereals and cereal-based products in the last decade.

Commodity	Area of Collection (Year)	AFs	Analytical Method	Contaminated Samples/Total Samples (Incidence %)	Mean (ppb)	Range (ppb)	Over EU MTL (%) *	References
Wheat	Punjab (2014)	AFB_1_	LCMS/MS	3/195 (2)	0.04	LOD–1.59	–	Xia et al. [116]
Wheat flour	Lahore (2018)	AFB_1_	TLC	10/30 (33.33)	2.197	1.83–3.01	30	Zahra et al. [117]
Cereal products	Local market, Punjab (2016)	AFB_1_ AFTs	HPLC HPLC	121/229 (53) 121/229 (53)	1.87 2.99	1.70–8.20 LOD–9.95	43 22	Alim et al. [118]
Wheat grains	Pakistan (2014)	AFB_1_ AFB_2_ AFTs	HPLC HPLC HPLC	48/185 (26) 13/185 (7) 48/185 (26)	0.51 0.02 0.53	0.05–4.78 0.02–0.48 0.02–5.26	45.8 0 10.4	Asghar et al. [119]
Wheat based products	Local market, Punjab	AFTs	HPLC	44/147 (30)	8.076	LOD–61.6	27.2	Iqbal et al. [120]
Non-branded rice	Punjab (2019)	AFTs	TLC, ELISA	65/100 (65)	3.51	0.75–8.92	27.6	Tahir et al. [121]
Unpacked rice	Punjab	AFB_1_	TLC, ELISA	12/50 (24)	6.98	LOD–20.1	58	Nazir et al. [122]
Rice	Punjab (2014)	AFB_1_	LCMS/MS	41/62 (66)	5.04	LOD–71.56	–	Xia et al. [116]
Rice	Local market, Punjab (2015)	AFB_1_ AFB_2_	LCMS/MS LCMS/MS	101/180 (56) 87/180 (48)	5.84 1.91	1.5–40 1.5–9.15	– –	Majeed et al. [123]
Brown rice Brown rice White rice White rice	Punjab (2010–2015)	AFTs AFB_1_ AFTs AFB_1_	TLC TLC TLC TLC	154/1081 (14.24) 854/1081 (79) 216/1170 (18.46) 896/1170 (76.58)	3.1 8.98 3.27 5.83	0.11–49.50 0.95–45 1.37–69.3 0.1–64.05	– – – –	Sultana et al. [124]
Super kernel (SK) basmati rice	Local markets, Punjab	AFTs AFB_1_ AFB_2_	HPLC HPLC HPLC	28/48 (58) 27/48 (56) 16/48 (33)	13.92 – –	2–26 0–27 –	93 100 –	Mukhtar et al. [125]
Brown rice	Local market	AFTs	TLC	23/25 (92)	–	–	60	Nisa et al. [126]
Rice and rice products	Local markets, Central Punjab (2012—2013)	AFB_1_ AFTs	HPLC HPLC	73/208 (35) 73/208 (35)	2.40 6.36	LOD–21.3 LOD–32.2	19 24	Iqbal et al. [127]
SK basmati rice SK basmati rice Basmati rice Parboiled rice Broken rice	City area, Lahore	AFB_1_ AFB_2_ AFB_1_ AFB_1_ AFB_1_	TLC, HPLC TLC, HPLC TLC, HPLC TLC, HPLC TLC, HPLC	48/361 (13.3) 6/361 (1.9) 107/585 (18.3) 30/70 (42.9) 4/11 (36.4)	– – – – –	1.1–32.9 1.0–8.1 1.0–15.4 1.1–9.2 2.1–25.3	6.4 0.3 6.0 10 36.4	Firdous et al. [128]
Brown rice	Karachi (2013)	AFB_1_ AFTs AFB_1_ AFTs	TLC HPLC LC-MS/MS ELISA	100/120 (83) 100/120 (83) 104/120 (87) 104/120 (87)	3.56 3.79 3.73 3.89	0.21–10.54 0.21–11.89 0.10–10.88 0.10–12.39	–	Iqbal et al. [41]
Brown rice	Vendors (2006—2011)	AFB_1_ AFB_2_ AFTs	TLC TLC TLC	250/262 (95.4) 20/262 (7.6) 250/262 (95.4)	3.80 0.09 3.89	1.07–24.65 0.52–2.62 1.07–27.27	23.68 0 23.68	Asghar et al. [129]
Export quality basmati rice	Vendors (2006—2011)	AFB_1_	TLC	1500/2047 (73.3)	1.15	1.17–6.91	2.74	Asghar et al. [130]
Rice	Retail markets, Punjab (2011—2012)	AFB_1_	HPLC	38/68 (56)	8.23	–	18	Majeed et al. [131]
Brown, white and parboiled rice	Export areas (2010)	AFB_1_ AFB_2_	HPLC HPLC	254/519 (48.9) 24/519 (4.6)	1.18 0.61	LOD–16.7 0.5–2.64	5.58 –	Firdous et al. [132]
White rice Brown rice Broken rice Paddy rice Parboiled rice	Retail markets, Fields, Punjab (2010)	AFTs AFTs AFTs AFTs AFTs	HPLC HPLC HPLC HPLC HPLC	39/93 (42) 28/84 (33) 55/109 (50) 37/58 (64) 26/69 (37)	7.10 9.85 8.50 16.35 14.20	LOD–58.2 LOD–28.7 LOD–38.5 LOD–68.3 LOD–39.4	48 43 90 40 73	Iqbal et al. [115]
Rice	(2008—2009)	AFB_1_ AFTs	TLC TLC	28/40 (70) 28/40 (70)	3.7 4.9	– –	50 –	Hussain et al. [108]
Maize	Northern areas, KPK	AFB_1_ AFB_2_	HP–TLC HP–TLC	9/10 (90) 5/10 (50)	27.87 1.35	9.09–48.48 0.05–3.75	– –	Murad et al. [133]
Branded corn Non-branded corn Non-branded corn Non-branded corn	Local market, Lahore (2016)	AFB_1_ AFB_1_ AFB_2_ AFG_1_	TLC TLC TLC TLC	75/75 (100) – 9/75 (12) 6/75 (8)	7.07 67.37 8.95 16.46	1.02–7.07 1.25–67.73 0–8.95 0–16.46	23 64 12 8	Hussain et al. [134]
Maize	(2016—2019)	AFB_1_ AFB_2_ AFG_1_ AFG_2_ AFTs	HPLC HPLC HPLC HPLC HPLC	267/324 (82.4) 20/324 (6.2) 13/324 (4.0) 7/324 (2.2) 267/324 (82.4)	17.76 0.50 0.20 0.07 18.53	0.69–315.32 1.35–25.65 0.98–11.41 0.94–4.50 0.69–356.72	66.6 0 0 0 56.2	Asghar et al. [88]
Maize	Faisalabad	AFTs	HPLC	41/46 (89)	29.8	27.4–362.8	100	Hassan et al. [135]
Yellow maize	Punjab	AFB_1_ AFB_2_ AFG_2_	HPLC HPLC HPLC	28/36 (77.7) 22/36 (61.1) 14/36 (38.8)	– – –	11–17 9–13 5–9	100 100 100	Manzoor et al. [136]
		AFB_1_ AFB_2_ AFG_2_	HPLC HPLC HPLC	20/36 (55.5) 13/36 (36.1) 7/36 (19.4)	– – –	7–13 2–6 1–6	100 – –	
Corn based product	City area, Lahore	AFB_1_ AFB_2_	HPLC HPLC	52/100 (52) 25/100 (25)	– –	2.0 –1405.3 1.0—55.2	52 –	Firdous et al. [128]
Maize	Punjab	AFB_1_ AFB_2_	HPLC HPLC	73/75 (97.3) 58/75 (78.9)	54.54 1.46	LOD–409.3 LOD–8.03	77.3 28	Iram et al. [137]
Corn Corn Corn products Corn products	Retail markets, local industries, Punjab (2011–2012)	AFB_1_ AFTs AFB_1_ AFTs	HPLC HPLC HPLC HPLC	37/105 (35.23) 37/105 (35.23) 43/102 (42.15) 43/102 (42.15)	7.90 12.08 5.47 7.85	– – – –	14 28 14 20	Majeed et al. [130]
Maize	Pakistan (2006–2007)	AFB_1_ AFB_2_ AFG_1_	HPTLC HPLC HPLC	18/65 (27.69) 12/65 (18.46) 2/65 (1.3)	192 40 9	5–850 3–187 8–11	27.69 18.46	Khatoon et al. [100]
Maize	Upper Swat, Lower Swat, KPK (2007)	AFB_1_ AFB_1_	HPLC HPLC	14/18 (77.78) 16/18 (88.89)	14.94 16.22	0–30.96 0–27.28	50 * 37 *	Shah et al. [138]
Maize	Urban areas Semi-urban Rural areas	AFTs AFTs AFTs	HPLC HPLC HPLC	(80) (87) (90)	45 54 62	– – –	– – –	Ahsan et al. [139]

Abbreviations (AFB_1_ = Aflatoxin B_1_, AFB_2_ = Aflatoxin B_2_, AFG_1_ = Aflatoxin G_1_, AFG_2_ = Aflatoxin G_2_, AFTs = Total aflatoxins, TLC = thin-layer chromatography, HPLC = high-performance liquid chromatography, LCMS = liquid chromatography mass spectroscopy, MS = mass spectroscopy, LC = liquid chromatography, HPTLC = high-performance thin-layer chromatography, KPK = Khyber Pakhtunkhwa, LOD = limit of detection, ppb = parts per billion, EU = European Union, MTL = maximum tolerance limit). (%) * = Percentage of the samples out of contaminated samples exceeding the maximum tolerance limits (MTL) recommended by European Union [98]. Value * exceeding the exceeding the MTL recommended by FAO and FDA [97].

**Table 2:** Occurrence of AFs in edible oilseeds and related products from 2011 to 2022.

Commodity	Area of Collection (Year)	AFs	Analytical Method	Contaminated Samples/Total Samples (Incidence %)	Mean (ppb)	Range (ppb)	Over EU MTL (%) *	References
Local edible seeds Local edible seeds Imported edible seeds Imported edible seeds	Local market, Punjab (2019)	AFB_1_ AFTs AFB_1_ AFTs	HPLC HPLC HPLC HPLC	108/189 (57) 108/189 (57) 92/162 (56.7) 92/162 (56.7)	13.551 21.17 9.21 15.59	– – – –	100 100 100 100	Waqas et al. [144]
Local edible oil Local edible oil Imported edible oil Imported edible oil	Local market, Punjab (2019)	AFB_1_ AFTs AFB_1_ AFTs	HPLC HPLC HPLC HPLC	103/213 (48.3) 103/213 (48.3) 78/180 (43.3) 78/180 (43.3)	8.83 14.52 5.04 9.34	– – – –	– – – –	Waqas et al. [144]
Peanut oil	Local market, Peshawar (2020—2021)	AFB_1_ AFB_2_ AFG_1_ AFTs	TLC TLC TLC TLC	42/60 (70) 31/60 (51.7) 2/60 (3.3) 42/60 (70)	6.20 3.40 4.90 8.59	0.12–25 0.2–22 2.4–7.4 0.12–55	41.7 – – 35	Hussain et al. [145]
Raw peanuts in shells Peanuts without shells Roasted peanuts R. P. without shells Peanuts butter Peanuts cookies Packed peanuts (nimko)	Local market, Punjab	AFTs AFTs AFTs AFTs AFTs AFTs AFTs	HPLC HPLC HPLC HPLC HPLC HPLC HPLC	13/22 (59) 16/29 (55) 19/31 (61) 13/19 (68) 16/32 (50) 10/24 (42) 8/41 (20)	6.4 9.6 10.4 12.3 2.4 4.6 3.4	LOD–59.8 LOD–82.1 LOD–71.3 LOD–119 LOD–32.2 LOD–31.8 LOD–11.4	32 17 29 36 25 13 –	Iqbal et al. [146]
Roasted peanut Salty peanuts Unripe peanuts	Pothohar plateau, Punjab	AFB_1_ AFB_1_ AFB_1_	HPLC HPLC HPLC	19/24 (79.25) 14/24 (58.5) 15/24 (62.5)	45.03 38.45 42.67	14–98 14–85 15–85	100 100 100	Abbas et al. [143]
Peanut (nimko)	Lahore and Faisalabad	AFTs	HPLC	3/5 (60)	–	0.21–2.08	–	Mushtaq et al. [147]
Peanuts with shell Peanut without shell	KPK and Northern areas (2009)	AFTs AFTs	LC LC	4/10 (40) 5/10 (50)	5.10 5.20	1.5–14.5 0.7–12.8	10 20	Lutfullah et al. [148]
Peanut and peanut products	Local market	AFB_1_ AFB_2_	TLC TLC	– –	66.48 7.71	5.0–183.2 7–46.7	– –	Mobeen et al. [103]
Sesame seeds	Punjab (2021)	AFB_1_ AFTs	HPLC HPLC	14/45 (31.1) 14/45 (31.1)	9.96 11.7	LOD–65.5 LOD–65.5		Iqbal et al. [149]
Sesame seeds	Rainfed (fresh) Rainfed (stored) Irrigated areas (fresh) Irrigated (stored) (2018)	AFB_1_ AFB_1_ AFB_1_ AFB_1_	TLC TLC TLC TLC	44/50 (88) 50/50 (100) 48/50 (96) 49/50 (98)	15.74 33.8 20.5 27.56	1.2–60 23–50 12–48 15–60	100 100 100 100	Ajmal et al. [150]

Abbreviations (AFB_1_ = Aflatoxin B_1_, AFB_2_ = Aflatoxin B_2_, AFG_1_ = Aflatoxin G_1_, AFG_2_ = Aflatoxin G_2_, AFTS = Total aflatoxins, TLC = thin-layer chromatography, HPLC = high-performance liquid chromatography, LC = liquid chromatography, KPK = Khyber Pakhtunkhwa, LOD = limit of detection, ppb = parts per billion, EU = European Union, MTL = maximum tolerance limit). (%) * = Percentage of the samples out of contaminated samples exceeding the maximum tolerance limits (MTL) recommended by European Union [98]. Value * exceeding the exceeding the MTL recommended by FAO and FDA [97].

**Table 3:** Occurrence of AFs in nuts and dried fruits from 2011 to 2020.

Commodity	Area of Collection (Year)	AFs	Analytical Method	Contaminated Samples/Total Samples (Incidence %)	Mean (ppb)	Range (ppb)	Over EU MTL (%) *	Reference
Nuts (winter) Nuts (summer)	Southern Punjab (2019)	AFTs AFTs	HPLC HPLC	180/414 (43.4) 122/365 (33.4)	15.43 12.71	0.05–50.5 0.05–45.6	11.5 * 12.2 *	Razis et al. [156]
Dry fruits Apricot kernel Apricot kernel Apricot kernel	Gilgit-Baltistan, Ghizer, Skardu	AFB_1_ AFB_1_ AFB_1_ AFB_1_	TLC TLC TLC TLC	75/300 (25) 7/50 (14) 10/50 (20) 13/50 (26)	5 2 3 3.5	0.5–15 0.5–4 1–6 0.5–5	40 28.57 30 30.76	Ali et al. [155]
Walnut Walnut Walnut		AFB_1_ AFB_1_ AFB_1_	TLC TLC TLC	11/50 (22) 15/50 (30) 19/50 (38)	4.5 6 8	0.6–10 0.6–13 0.6–15	45.5 40 47.37	
Nuts	Punjab and KPK (2016– 2017)	AFTs	HPLC	128/320 (40)	4.8	LOD–20.70	34.6	Iqbal et al. [146]
Apricot kernels Almonds with shell Almonds without shell Walnuts with shell Walnuts without shell	Local market, KPK, and northern areas (2009)	AFTs AFTs AFTs AFTs AFTs	LC LC LC LC LC	4/15 (26.67) 0/10 (0) 3/10 (30) 4/10 (40) 7/10 (70)	2.65 – 2.13 6.45 3.43	0.7–5.6 – 1.2–3.4 1.5–13.5 0.8–10.8	25 – – 50 42.85	Lutfullah et al. [148]
Pistachios with shell Without shell Pine nuts with shell	Local market, KPK, and northern areas (2009)	AFTs AFTs AFTs	LC LC LC	2/10 (20) 5/10 (50) 2/10 (20)	2.10 6.34 3.25	1.2–3.0 2.0–14.0 2.6–3.9	– 40 –	Lutfullah et al. [148]

Abbreviations (AFB_1_ = Aflatoxin B_1_, AFTs = Total aflatoxins, TLC = thin-layer chromatography, HPLC = high-performance liquid chromatography, LC = liquid chromatography, KPK = Khyber Pakhtunkhwa, LOD = limit of detection, ppb = parts per billion, EU = European Union, MTL = maximum tolerance limit). (%) * = Percentage of the samples out of contaminated samples exceeding the maximum tolerance limits (MTL) recommended by European Union [98]. Value * exceeding the exceeding the MTL recommended by FAO and FDA [97].

**Table 4:** Occurrence of AFs in spices and related products from 2007 to 2021.

Commodity	Area of Collection (Year)	AFs	Analytical Method	Contaminated Samples/Total Samples (Incidence %)	Mean (ppb)	Range (ppb)	Over EU MTL (%) *	Reference
Red chili sauce Red chili sauce Green chili sauce Green chili sauce Red chili garlic sauce Red chili garlic sauce	Dhaba, local restaurants, and fast-food outlets, Punjab (2018)	AFB_1_ AFTs AFB_1_ AFTs AFB_1_ AFTs	LC LC LC LC LC LC	65/87 (74.71) 65/87 (74.71) 60/80 (75) 60/80 (75) 64/85 (75) 65/87 (74.71)	9.7 23.86 4.43 15.76 4.36 18.86	LOD–115.5 LOD–120 LOD–71.20 LOD–78 LOD–85.5 LOD–90	– – – – – –	Iqbal et al. [168]
Dried red chilies	Kunri, Sindh (2012)	AFB_1_ AFB_2_ AFG_1_ AFTs	TLC, HPLC TLC, HPLC TLC, HPLC TLC, HPLC	46/69 (67) 46/69 (67) 1/69 (1.44) 46/69 (67)	131.7 108.98 0.4 243.21	1.2–600 – – –	– – – –	Akhund et al. [169]
Packed spices Unpacked spices	Local market (2014)	AFTs AFTs	HPLC HPLC	49/100 (49) 74/100 (74)	4.789 26.34	– –	– –	Naz et al. [170]
Unpacked composite spices	Local market, Karachi, (2015)	AFTs	HPLC	58/75 (77)	4.63	0.68–25.74	3 *	Asghar et al. [171]
Whole red chilies Powder Crushed	Warehouses, supermarkets, and shops (2006–2011)	AFTs AFTs AFTs	TLC TLC TLC	223/226 (98.7) 64/69 (92.8) 33/36 (94.4)	11.7 27.8 31.2	1.3–91.4 5.1–77.9 1.6–93.7	9.7 * 52.8 * 52.8 *	Khan et al. [172]
Spices	Local market, Peshawar	AFTs	TLC	12/18 (66.67)	2.86	1.86–6.54	58	Hussain et al. [102]
Whole chilies Powdered chilies	Faisalabad (2008)	AFB_1_ AFB_1_	HPLC HPLC	16/22 (73) 19/22 (86.4)	32.20 26.64	0.00–89.56 0.00–96.3	– –	Iqbal et al. [173]
Dried chilies	Local market	AFTs	HPLC	13/13 (100)	33.43	0.1–96.2	76.92	Paterson [174].

Abbreviations (AFB_1_ = Aflatoxin B_1_, AFB_2_ = Aflatoxin B_2_, AFG_1_ = Aflatoxin G_1_, AFG_2_ = Aflatoxin G_2_, AFTS = Total aflatoxins, TLC = thin-layer chromatography, HPLC = high-performance liquid chromatography, LC = liquid chromatography, LOD = limit of detection, ppb = parts per billion, EU = European Union, MTL = maximum tolerance limit). (%) * = Percentage of the samples out of contaminated samples exceeding the maximum tolerance limits (MTL) recommended by European Union [98]. Value * exceeding the exceeding the MTL recommended by FAO and FDA [97].

**Table 5:** Occurrence of Afs in feed from 2012 to 2022.

Commodity	Area of Collection (Year)	Afs	Analytical Method	Contaminated Samples/Total Samples (Incidence %)	Mean (ppb)	Range (ppb)	Over EU MTL (%) *	References
Poultry feed	Punjab (2019)	AFB_1_	LC-MS	150/150 (100)	39.9	–	73	Sarwat et al. [186]
Poultry feed samples	Northern Pakistan (2018)	AFB_1_	ELISA	37/40 (92.5)	54.56	–	–	Naveed et al. [187]
Poultry feed	Baluchistan	AFB_1_	ELISA	77/100 (77)	–	–	–	Majeed et al. [188]
Cattle feed	Punjab	AFB_1_	TLC and ELISA	25/60 (41.66)	15.59	1.9–28.5	12	Nazir et al. [122]
Animal feed	Punjab, Pakistan (2017–2018)	AFB_1_	LC	126/193 (65.3%)	25.0	–	45.6	Waqas et al. [189]
Cotton seeds Cotton seeds cake	Karachi and Hyderabad	AFB_1_	ELISA and HPLC	88/110 (80) 97/110 (88)	69 89	– –	64 71	Shar et al. [190]
Bovine feed	Lahore	AFB_1_ AFB_2_ AFTs	TLC TLC TLC	30/50 (60) 10/50 (20) 30/50 (60)	214.9 46.4 229.7	24.1–361.4 4.5–81.3 28.6–394.3	– – –	Summia et al. [185]
Animal feed	Dairy farms, Punjab (2015)	AFB_1_	ELISA	(53)	43.15	8–119	95 *	Akbar et al. [191]
Mixed layer poultry feed	Karachi (2015–2016)	AFB_1_	ELISA	132/132 (100)	25.51	4.23–72.27	31	Iram et al. [192]
Commercial feed Fresh fodder Leftover bread	Punjab (2014–2015)	AFB_1_ AFB_1_ AFB_1_	ELISA ELISA ELISA	22/72 (30.5) 2/72 (2.8) 62/72 (88.9)	4.92 3.04 6.72	1.04–9.76 0.64–4.61 3.96–11.34	30.5 2.8 88.9	Ismail et al. [193]
Animal feed	Farmers, industry, and general stores, Punjab (2012–2013)	AFB_1_ AFTs	HPLC HPLC	69/105 (66) 69/105 (66)	4.71 6.84	0.09–145.7 LOQ–165.5	– –	Iqbal et al. [194]
Feed ingredients Dairy feed	Lahore (2013–2014)	AFB_1_ AFB_1_	ELISA ELISA	54/125 (43) 41/90 (45.56)	36.53 16.54	1.02–210.07 0.56–55.17	– –	Chohan et al. [195]
Poultry feed ingredient Poultry feeds	Various parts of country (2009–2010)	AFB_1_ AFB_1_	TLC TLC	46/77 (60) (44.39)	37.62 23.75	LOD–56 LOD–78	– –	Anjum et al. [183]
Poultry feed	Broiler farms (2009–2010)	AFB_1_	TLC	88/96 (91.66)	47.64	10–166	82.30 *	Rashid et al. [179]

Abbreviations (AFB_1_ = Aflatoxin B_1_, AFB_2_ = Aflatoxin B_2_, AFTS = Total aflatoxins, TLC = thin-layer chromatography, HPLC = high-performance liquid chromatography, LCMS = liquid chromatography mass spectroscopy, MS = mass spectroscopy, LOD = limit of detection, ppb = parts per billion, EU = European Union, MTL = maximum tolerance limit). (%) * = Percentage of the samples out of contaminated samples exceeding the maximum tolerance limits (MTL) recommended by European Union [98]. Value * exceeding the exceeding the MTL recommended by FAO and FDA [97].

## Data Availability

The data presented in this study are available in this article.
